# Supplementary material for: Simultaneous Integration of Gene Expression and Nutrient Availability for Studying the Metabolism of Hepatocellular Carcinoma Cell Lines
Source: Biomolecules. 2021 Mar 24;11(4):490. doi: 10.3390/biom11040490 (PMC8064315; doi:10.3390/biom11040490)
Supplement: Supplementary file 1 [file biomolecules-11-00490-s001.pdf]

Supporting Information
for

Simultaneous Integration of Gene Expression and Nutrient Availability for Studying the Metabolism of Hepatocellular Carcinoma Cell Lines

Ewelina Węglarz-Tomczak[1*], Thierry D.G.A. Mondeel[1], Diewertje G.E. Piebes[1], Hans V. Westerhoff[1,2,3]

[1]Swammerdam Institute for Life Sciences, Faculty of Science, University of Amsterdam, Amsterdam, The Netherlands
[2]Molecular Cell Physiology, Amsterdam Institute for Molecules, Medicines and Systems, Faculty of Science, Vrije Universiteit Amsterdam, Amsterdam, The Netherlands
[3]Manchester Centre for Integrative Systems Biology, School for Chemical Engineering and Analytical Sciences, University of Manchester, Manchester, UK

*Corresponding author
E-mail: ewelina.weglarz.tomczak@gmail.com

S1. Supplementary figures

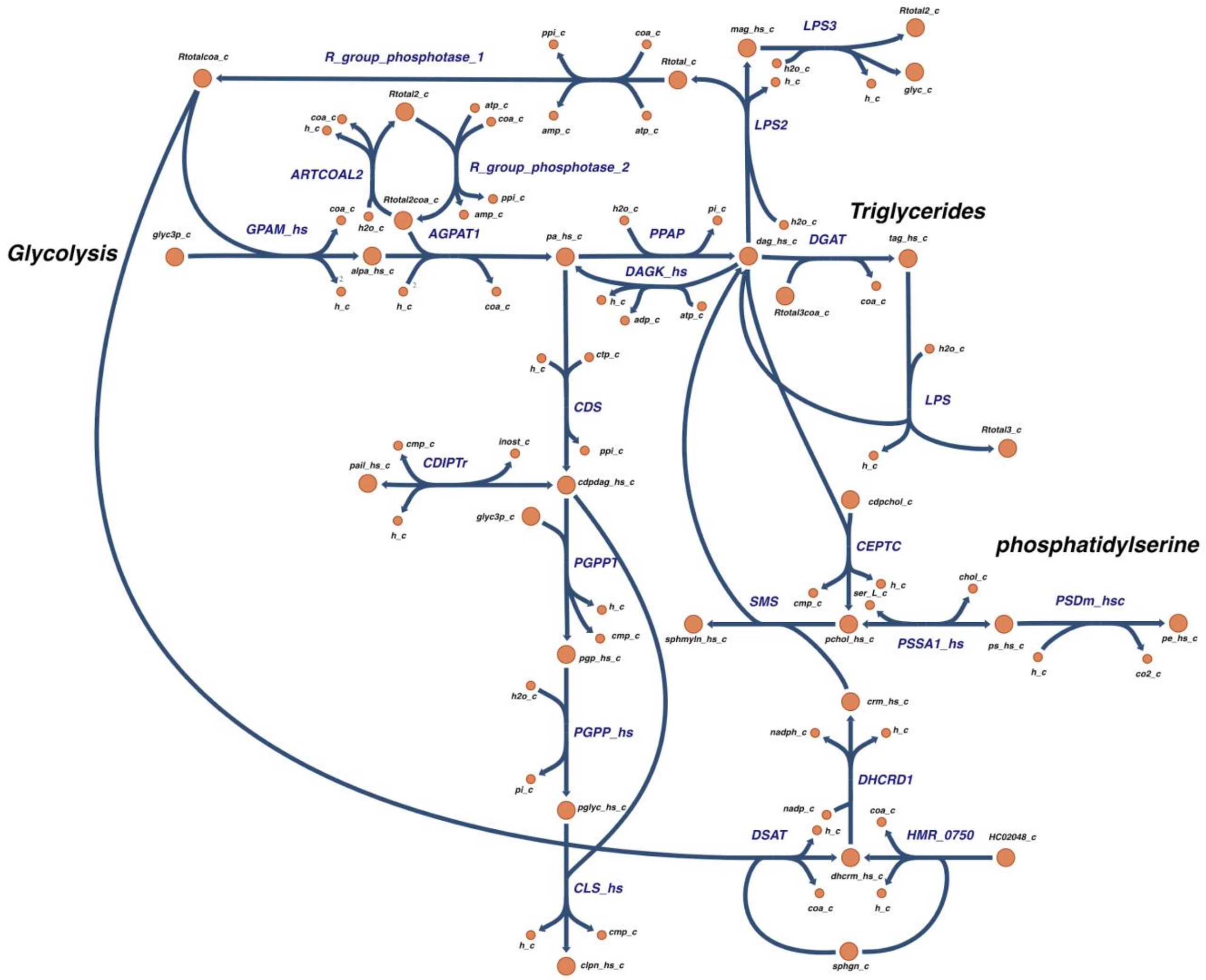

Figure S1. Network diagram of the subnetwork of the published version of Recon3.01 related to lipid synthesis from glycerol-3-phosphate (referred to as *glyc3p_c*) drawn using Escher [1]. See Figure S2 for the updated network diagram.

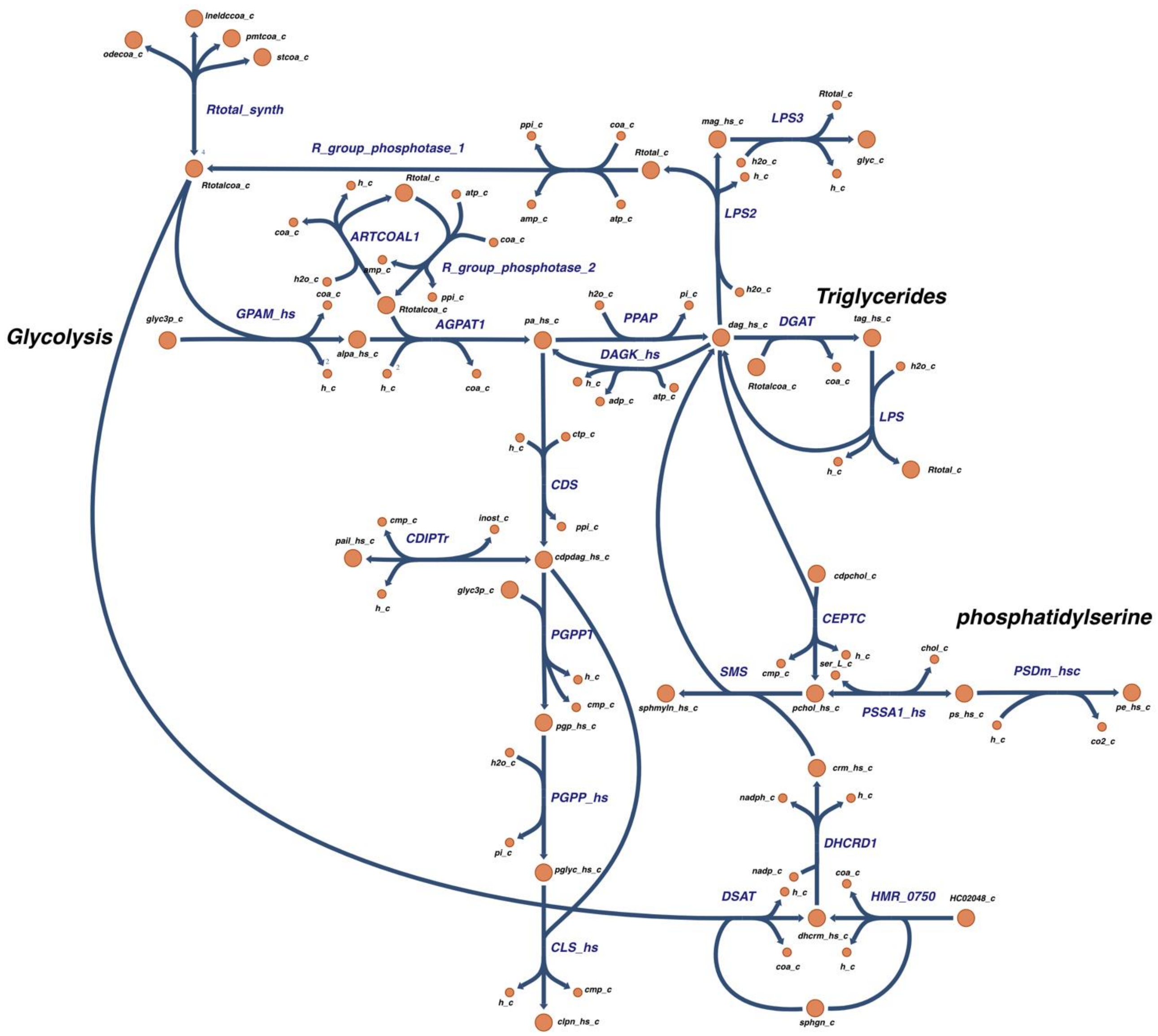

Figure S2. Network diagram of the updated subnetwork in Recon3.01 related to lipid synthesis from glycerol-3-phosphate (referred to as *glyc3p_c*) drawn using Escher [1]. We enhanced the pathway by adding a synthesis reaction for Rtotal and by equating the various forms of Rtotal by adding reversible reactions between them (see main text).

A

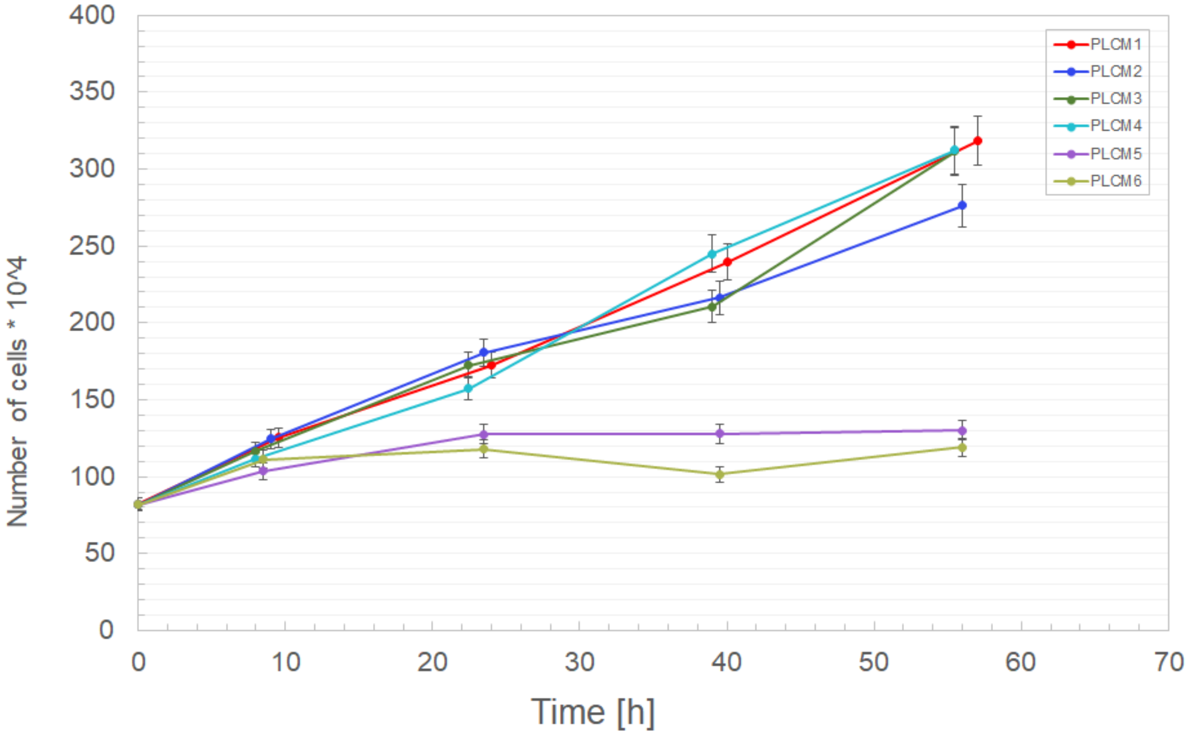
400
350
300
250
200
150
100
50
0
Number of cells * 10^4
0
10
20
30
40
50
60
70
Time [h]
PLCM1
PLCM2
PLCM3
PLCM4
PLCM5
PLCM6

B

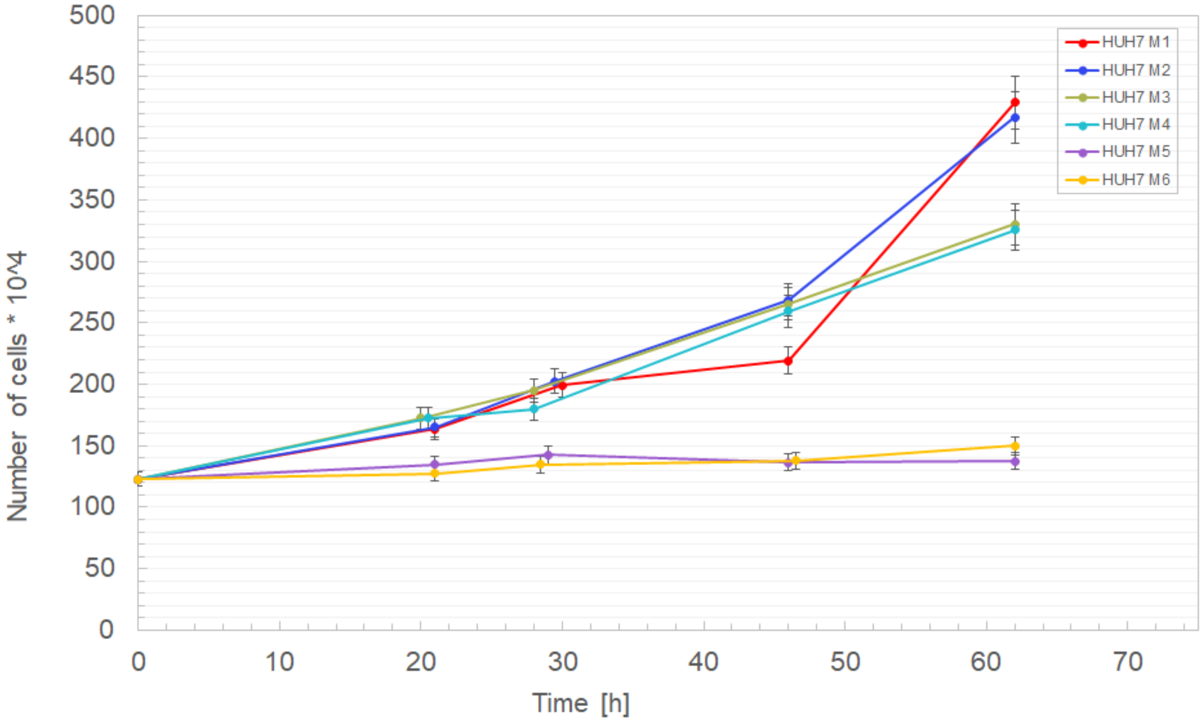
500
450
400
350
300
250
200
150
100
50
0
Number of cells * 10^4
0
10
20
30
40
50
60
70
Time [h]
HUH7 M1
HUH7 M2
HUH7 M3
HUH7 M4
HUH7 M5
HUH7 M6

C

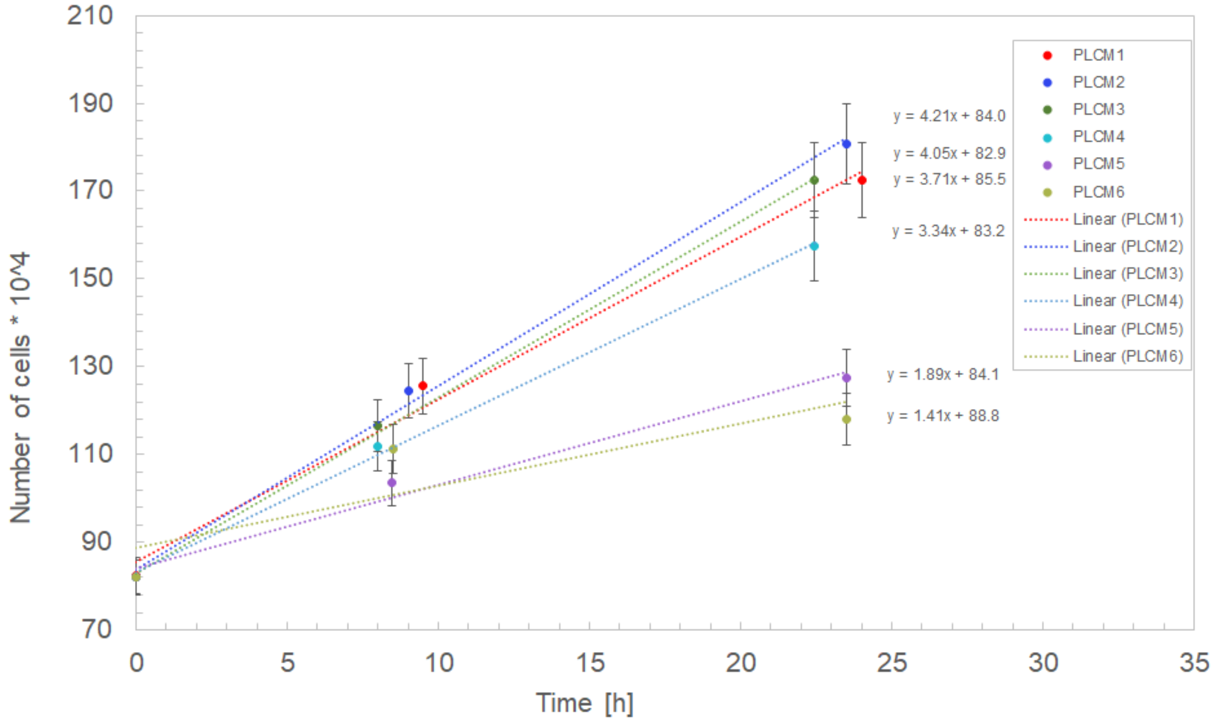

D

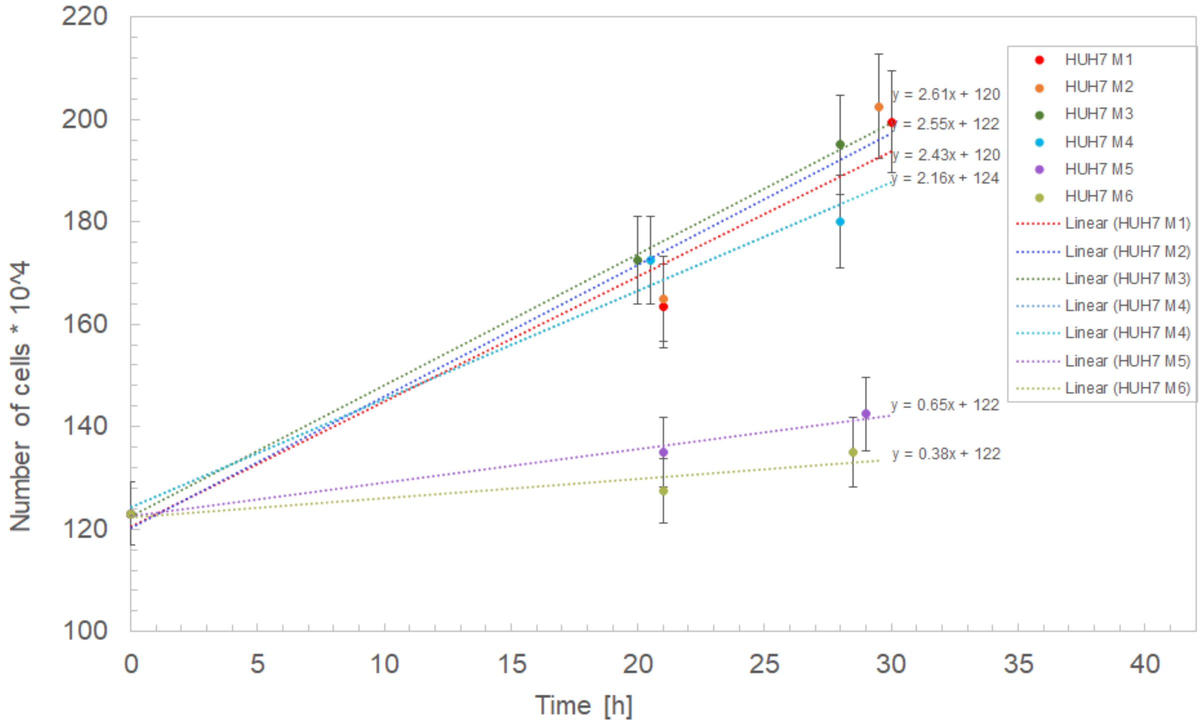

Figure S3. The growth rate of PLC (**A** and **C**) and Huh7 (**B** and **D**) cells in different media for the entire experiment (**A** and **B**) and the first 24 hours (**C** and **D**) that were used to calculate the linear relationship. The Y-axis represents the number of cells, the X-axis represents the time expressed in hours.

S2. Supplementary tables

Table S1. List of Entrez identifiers for genes present in Recon3D that we were not able to identify in the RNA-seq dataset by Ma et al. after converting the Entrez ID to the respective gene symbol and possible aliases. Red cells concern Entrez identifiers that do not exist, whereas orange cells concern Entrez identifiers that have been withdrawn from NCBI. The 16 genes in white or orange cells were assumed to be expressed at excess levels.

Entrez ID	Symbol	Alias	Name
201288.1	NOS2P2	NOS2B	nitric oxide synthase 2 pseudogene 2
645740.1	NOS2P1	NOS2C	nitric oxide synthase 2 pseudogene 1
728441.1	GGT2	GGT, GGT 2	gamma-glutamyltransferase 2
102724560.1	CBSL	CBS	cystathionine-beta-synthase like
65263.1	PYCR3	PYCRL	pyrroline-5-carboxylate reductase 3
8781.1	PSPHP1	CO9, PSPHL	phosphoserine phosphatase pseudogene 1
100507855.1			
644378.1	GCNT2P1	GCNT2P, GCNT6	GCNT2 pseudogene 1
284004.1	HEXD	HEXDC	hexosaminidase D
8041.1			
100288072.1	SDR42E2		short chain dehydrogenase/reductase family 42E, member 2
340811.1	AKR1C8P	AKR1CL1	aldo-keto reductase family 1 member C8, pseudogene
0			
4514.1	COX3	COIII, MTCO3	cytochrome c oxidase III

4513.1	COX2	COII, MTCO2	cytochrome c oxidase subunit II
4512.1	COX1	COI, MTCO1	cytochrome c oxidase subunit I

Table S2. Metabolic components of the biomass reaction in Recon3D listed along with the stoichiometry at which they were taken to contribute to the biomass pseudo reaction. The stoichiometric coefficient is negative if the compound was taken to be consumed in the biomass synthesis reaction and positive if it was produced therein. It has been obtained [2] by determining the mass-fractional contributions of each biomass precursor to the dry weight of the biomass (i.e. in terms of milligram precursor per gram of dry weight (DW)). This fraction is then divided by the molecular weight of that precursor to obtain the above coefficient, which then is in unit mmol precursor per gram of biomass dry weight (g DW). The biomass reaction equation is then formulated as the sum of each metabolite precursor multiplied by its above coefficient. The biomass reaction rate is the specific growth rate and has units (g DW / g DW)/h. The specific consumption rates of the metabolites are obtained by multiplying their above coefficients by the specific growth rate and thereby are in units mmol/(g DW)/h [3]. Multiplying these specific consumption rates by the biomass concentration in g DW/dm^3 of the culture vessel, one obtains the consumption flux in terms of mM/h.

**Metabolite**	**Coefficient**
Adenosine Diphosphate	20.65082
L-Alanine	-0.50563
L-Arginine	-0.35926
L-Asparagine	-0.27943
L-Aspartate	-0.35261
Adenosine Triphosphate	-20.7045
Cholesterol	-0.0204

Cardiolipin	-0.01166
Cytidine-5'-Triphosphate	-0.03904
L-Cysteine	-0.04657
Deoxyadenosine Triphosphate	-0.01318
Deoxycytidine-5'-Triphosphate	-0.00944
Deoxyguanosine-5'-Triphosphate	-0.0099
Deoxythymidine-5'-Triphosphate	-0.01309
D-Glucose 6-Phosphate	-0.27519
L-Glutamine	-0.326
L-Glutamate	-0.38587
Glycine	-0.53889
Guanosine-5'-Triphosphate	-0.03612
Water	-20.65082
Proton	20.65082
L-Histidine	-0.12641
L-Isoleucine	-0.28608
L-Leucine	-0.54554
L-Lysine	-0.59211
L-Methionine	-0.15302
1-Phosphatidyl-1D-Myo-Inositol	-0.02332

Phosphatidylcholine	-0.15446
Phosphatidylethanolamine	-0.05537
Phosphatidylglycerol	-0.00291
L-Phenylalanine	-0.25947
Orthophosphate	20.65082
L-Proline	-0.41248
Phosphatidylserine	-0.00583
L-Serine	-0.39253
Sphingomyelin	-0.01749
L-Threonine	-0.31269
L-Tryptophan	-0.01331
L-Tyrosine	-0.15967
Uridine-5'-TrIphosphate	-0.05345
L-Valine	-0.35261

Table S3. List of the oxidative phosphorylation reactions considered in Figure 5 by their reaction ID in Recon3D and with their reaction as specified in Recon3D.

**ID**	**Name**	**Reaction**
ATPS4mi	ATP synthase	adp_m + 4.0 h_i + pi_m --> atp_m + h2o_m + 3.0 h_m
NADH2_u10mi	NADH dehydrogenase	5.0 h_m + nadh_m + q10_m --> 4.0 h_i + nad_m + q10h2_m

CYOR_u10mi	ubiquinol-cytochrome c reductase	2.0 ficytC_m + 2.0 h_m + q10h2_m --> 2.0 focytC_m + 4.0 h_i + q10_m
CYOOm2i	cytochrome c oxidase	4.0 focytC_m + 8.0 h_m + o2_m --> 4.0 ficytC_m + 2.0 h2o_m + 4.0 h_i
CYOOm3i	cytochrome c oxidase	4.0 focytC_m + 7.92 h_m + o2_m --> 4.0 ficytC_m + 1.96 h2o_m + 4.0 h_i + 0.02 o2s_m
PDHm	pyruvate dehydrogenase	coa_m + nad_m + pyr_m --> accoa_m + co2_m + nadh_m

Table S4. Concentrations of metabolites in the DMEM media in mM as listed in the manufacturer's formulation. Our media M1-M6 only differ in the glucose and glutamine concentrations (colored in green) and were exempt of ammonia. All concentrations were effected in-silico as maximal uptake rates which shape and constrain the flux cone of the solutions in FBA (see text). For carbon dioxide and oxygen, a not limiting uptake bound of 1000 was taken. Oxygen was considered non-limiting because the concentration in the medium was in the order of 0.2-0.4 mM (corresponding to air saturated saline) [4] whereas the K_m of cytochrome oxidase for oxygen is some 0.01 mM [5]. Carbon dioxide was considered non-limiting due to its continuous replenishment in the medium.

**Metabolite**	**Uptake bound**	**Metabolite**	**Uptake bound**	**Metabolite**	**Uptake bound**	**Metabolite**	**Uptake bound**
Arginine	0.40	Bicarbonate	44	Nicotinamide	0.033	Thiamin	0.011
Choline	0.028	Histidine	0.2	Valine	0.80	Threonine	0.80
Cysteine	0.20	Isoleucine	0.80	Phenylalanine	0.4	Tryptophan	0.078
Iron (Fe3+)	0.00024	Myo-Inositol	0.04	Phosphate	0.9	Tyrosine	0.40
Folate	0.0091	Potassium	5.3	(R)-Pantothenate	0.0083	Carbon dioxide	1000
Glycine	0.40	Methionine	0.20	Serine	0.40	Oxygen	1000
Water	[54220,	Sodium	155	Sulfate	0.81		

	54250, 54100, 54440, 54470, 54500]						
Glucose	[0,5.6,25]	Leucine	0.80	Pyridoxine	0.019		
Glutamine	[0, 4.0]	Lysine	0.80	Riboflavin	0.0011		

References:

1. King ZA, Dräger A, Ebrahim A, Sonnenschein N, Lewis NE et al. Escher: A Web Application for Building, Sharing, and Embedding Data-Rich Visualizations of Biological Pathways. PLOS Comp Biol. 2015; 11(8): e1004321.
doi: 10.1371/journal.pcbi.1004321.

2. Brunk E, Sahoo S, Zielinski DC, Altunkaya A, Dräger A et al. Recon3D enables a three-dimensional view of gene variation in human metabolism. Nat Biotechnol. 2018; 36(3): 272-281.
doi: 10.1038/nbt.4072.

3. Thiele I, Palsson BØ. A protocol for generating a high-quality genome-scale metabolic reconstruction. Nat Protoc. 2010; 5(1): 93-121.
doi: 10.1038/nprot.2009.203.

4.Place TL, Domann FE, Case A J. Limitations of oxygen delivery to cells in culture: An underappreciated problem in basic and translational research. Free Radic Biol Med. 2017; 113: 311–322.
Google Scholar

5.↵Krab K, Kempe H. Wikström M. Explaining the enigmatic KM for oxygen in cytochrome c oxidase: A kinetic model. Biochim Biophys Acta. 2011; 1807(3): 348–358. doi: 10.1016/j.bbabio.2010.12.015.
